# An object detection algorithm combining self-attention and YOLOv4 in traffic scene

**DOI:** 10.1371/journal.pone.0285654

**Published:** 2023-05-18

**Authors:** Kewei Lu, Fengkui Zhao, Xiaomei Xu, Yong Zhang

**Affiliations:** College of Automobile and Traffic Engineering, Nanjing Forestry University, Nanjing, 210037, China; Menoufia University, EGYPT

## Abstract

Automobile intelligence is the trend for modern automobiles, of which environment perception is the key technology of intelligent automobile research. For autonomous vehicles, the detection of object information, such as vehicles and pedestrians in traffic scenes is crucial to improving driving safety. However, in the actual traffic scene, there are many special conditions such as object occlusion, small objects, and bad weather, which will affect the accuracy of object detection. In this research, the SwinT-YOLOv4 algorithm is proposed for detecting objects in traffic scenes, which is based on the YOLOv4 algorithm. Compared with a Convolutional neural network (CNN), the vision transformer is more powerful at extracting vision features of objects in the image. The CNN-based backbone in YOLOv4 is replaced by the Swin Transformer in the proposed algorithm. The feature-fusing neck and predicting head of YOLOv4 is remained. The proposed model was trained and evaluated in the COCO dataset. Experiments show that our method can significantly improve the accuracy of object detection under special conditions. Equipped with our method, the object detection precision for cars and person is improved by 1.75%, and the detection precision for car and person reach 89.04% and 94.16%, respectively.

## Introduction

Intelligent cars have been promised for many attractive features, such as automatic obstacle avoidance, vehicle cruise, and self-driving, based on on-board sensors, car control algorithms, and other technologies [1,2]. Intelligent cars effectively improve road safety and driving efficiency [3,4].

Therefore, an intelligent driving system is one of the important research directions in the automotive industry today. The intelligent driving system mainly includes three modules: environmental perception, autonomous decision-making, and motion control. Among them, environmental perception is the key technology in the research of the intelligent driving system, and it is also a prerequisite for cars to achieve autonomous driving. Therefore, it has great research significance and value for the research and application of object detection technology in traffic scenes. However, object detection is always a challenging task in situations where traffic conditions are complex.

Traditional object detection algorithms are based on hand-designed complex feature representation methods. For example, P. Viola and M. Jones proposed the VJ detector [5]; Dalal N et al. proposed an object detection algorithm based on the directional gradient histogram [6]; Felzenszwalb P F proposed a discriminatively trained, multiscale, deformable part model, which was extended based on the Histograms of Oriented Gradient (HOG) detector to improve detection performance [7]. However, the defects of the traditional object detection algorithm are as follows: On the one hand, the active window is used to detect the object. The number of windows is large and there is no persistence, which leads to the high complexity of the algorithm. On the other hand, the accuracy and generalizability of the traditional object detection algorithm needs to be improved.

Deep learning is playing important roles in different areas. In the field of transportation, deep learning can be used to achieve object detection in traffic scenes [8] and lane detection in intelligent driving [9]. Deep learning can help realize climate change forecast [10], air pollution classification and forecast [11], detection or classification of botnets [12]. What’s more, the supervised image classification algorithm is used to realize the classification of forest areas [13] and Convolutional Neural Network is applied to automatic weed detection system [14]. Deep learning is also widely used in fault diagnosis and remaining useful life prediction of mechanical equipment [15–18]. Deep learning is a learning method based on deep artificial neural networks and has predominant role in computer vision. At present, there are many excellent convolution-based deep learning object detection algorithms, such as two-stage object detection algorithms Mask R-CNN [19], SPP-Net [20], FOCS [21], Faster R-CNN [22,23] et al. Another single-stage object detection algorithms, such as YOLO [24–27] series, RetinaNet [28] et al. The deep convolutional network has good feature representation and classification ability, which is widely used in image classification, object detection, scene classification and other visual tasks, and has high accuracy [29]. At the same time, the deep learning-based object detection algorithm can adaptively construct feature descriptions driven by training data, solving the problem of difficulty of object detection caused by the changeable appearance of vehicles and has higher flexibility. Nevertheless, changes in weather or light, object occlusion, and hybrid background may affect the accuracy of object detection, leading to the decrease of accuracy and detection speed, which may result in the occurrence of traffic accidents.

In this paper, improving the quality of feature layer is regarded as a breakthrough point. We are devoted to detecting “person” and “car” of instances within images from the COCO2017 dataset with the proposed object detection algorithm at the goal of better experimental results. The main contributions are that the algorithm uses the Swin Transformer model as the backbone network, combined with the feature fusion module of YOLOv4, and uses the SPP-Expand the sensory wild network and the PANet- Feature fusion network to obtain a higher-quality feature layer, and improves the precision of object detection in traffic scenes with complex and changeable conditions, bad weather, fuzzy objects and small objects.

The rest of this paper is organized as follows. First of all, we briefly introduce the development of object detection approaches and the principles on which our proposed approach is based. Then we give the details and overall process in our proposed model. Next, we presented the results and analyses inferred from experiments conducted on COCO2017 datasets. Finally, we put forward the conclusion and make the prospect of the research.

## Related work

As one significant branch of computer vision, the object detection algorithm is the core of environment perception system, which plays an important role in the field of automatic driving. During the last decade, object detection algorithms based on deep learning have achieved many breakthroughs. The mainstream detection framework could be generally divided into two types: tow-stage and one-stage framework. The former generates region generates region proposals as sample at first and then classifies each sample into different object categories with the convolutional neural network while the latter directly turns the object box location problem into a regression problem to obtain accurate locations. Generally, one-stage object detection algorithms have better real-time performance, but lower accuracy, while two-stage algorithms have better accuracy, but weaker real-time performance.

Following the emerging trend of exploring deep learning, object detection algorithms have been continuously extended. In 2020, Hurtik P et al. proposed a Poly-YOLO algorithm [30]. The algorithm based on the YOLOv3 algorithm and uses a lightweight SE-Darkent-53 feature extraction network, which increases mAP by 40% and doubles detection speed; In 2021, Z Jin, P Qu et al. proposed a new algorithm to improve helmet wear detection of YOLOv5 [31]. They use the K-means++ algorithm to improve the degree of size matching of the a priori anchor box. Then integrate the Depthwise Coordinate Attention (DWCA) mechanism into the backbone network to strengthen the network’s ability to distinguish foreground and background; In 2022, Anlin Zheng and Yuang Zhang et al. proposed a new query-based detection framework for crowd detection [32]. They first select accepted queries prone to generate true positive predictions, and then refine the rest noisy queries according to the previously accepted prediction. The experimental results show that in the case where the approach is used, Spare RCNN achieves 92.0% AP; T Zhang and Y Huang et al. proposed the Cross Layer Refinement Network (CLRNet) with the aim of fully utilizing high and low-level features in lane detection [33]. The network detects lanes with high-level semantic features and then performs refinement based on low-level features in order to exploit more contextual information to detect lanes while leveraging local detailed lane features to improve localization accuracy. Experiments demonstrate that the proposed method outperforms state-of-the-art lane detection approaches. Huang CC, Chen SQ, Xu LT proposed a novel object detection method which is based on multi-source information fusion to deal with the low accuracy of object detection in different traffic scenes [34]. The method first adopts a traditional two-stage detection network to extract visual features, then extracts semantic features and relation features between objects, finally designing a multi-source information fusion module to integrate these features above; Wang C Y, Bochkovskiy A, Liao H proposed the YOLOv7 algorithm [35]. They design several trainable bag-of-freebies methods and propose ‘extend’ and ‘compound scaling’ methods for the real-time object detector that can efficiently utilize parameters and computation. Experiments showed that this method has a faster inference speed and higher detection accuracy. However, the detection accuracy of the current object detection algorithm for object occlusion, small objects, and bad weather in traffic scenes still needs to be improved. The precision of target detection can be enhanced by strengthening the quality of the feature layer extracted by the backbone network in the target detection algorithm and paying more attention to the fusion of multi-source information.

To sum up, it is always a challenging task for object detection algorithm to maintain high accuracy and real-time performance in the face of complex and changeable traffic scenes, especially fuzzy objects, small objects and bad weather. Specifically, the self-attention mechanism is introduced in our algorithm. The algorithm uses the Swin Transformer model as the backbone network, combined with the feature module of YOLOv4 and uses the SPP network and PANet network to obtain a higher-quality feature layer, thus optimizing the model for better training and test results.

## Materials and methods

### Overall structure of the swin transformer

**Fig 1:**
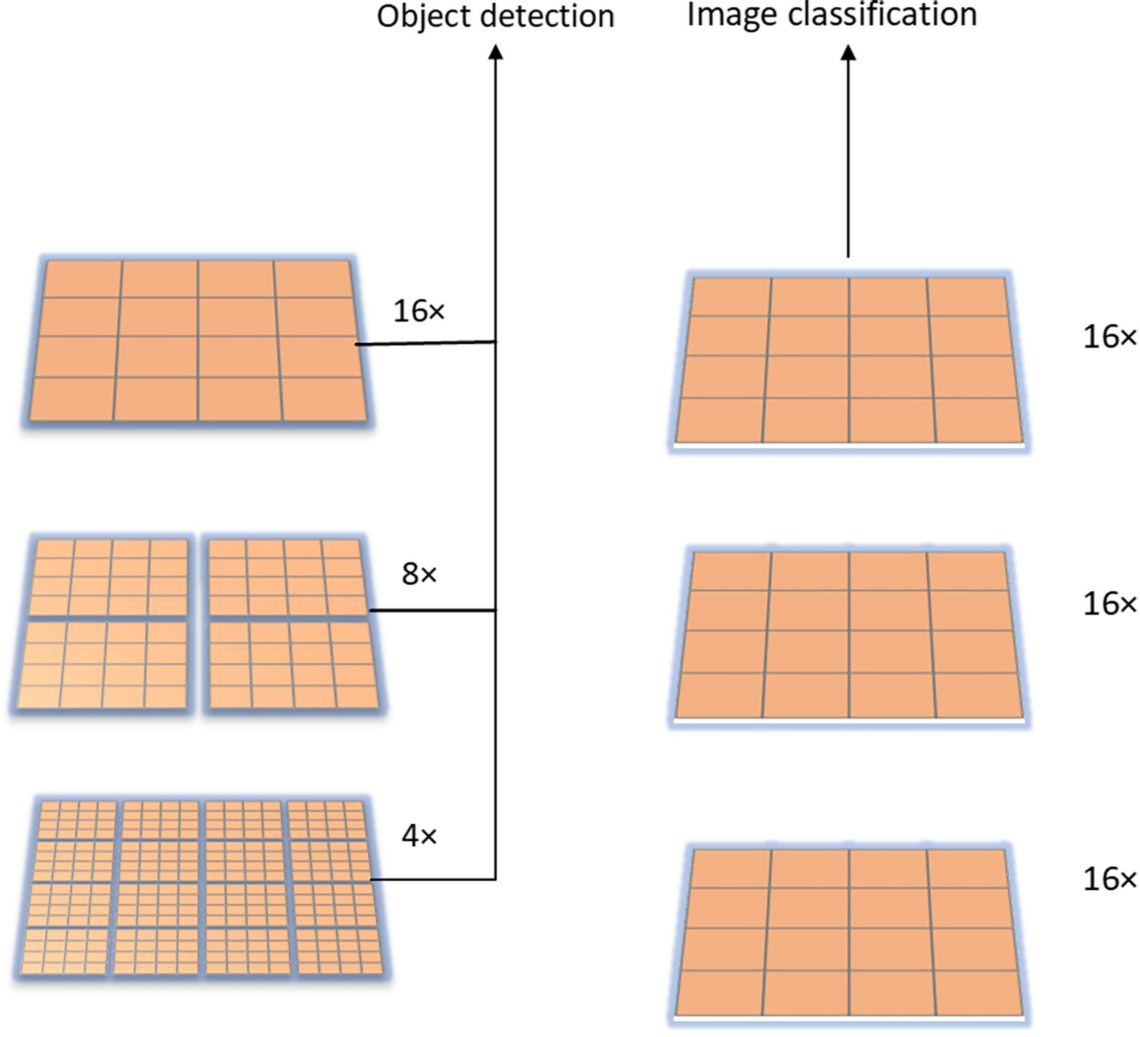
Comparison of the Swin Transformer and the Vision Transformer. (A) Swin Transformer; (B)Vision Transformer.

Transformer is a deep neural network based mainly on self-attention mechanism, which was initially widely used in natural language processing tasks and achieved great success in the language field [36]. Therefore, researchers began to study its adaptability to computer vision tasks and gradually extended it to computer vision tasks. In 2020, Google proposed the ViT (Vision Transformer) model based on the Transformer architecture and applied it to the image classification task [37]. After pretraining on a large-scale dataset, the best model obtained was in ImageNet, which can achieve 88.55% accuracy. However, when the ViT model extracts image features, the feature extraction of each feature layer is performed at a down-sampling rate of 16 times, and the image block size of each layer is fixed, while the image changes frequently in the visual field. Therefore, it is difficult to adapt to the extraction of feature values from widely varying object sizes, which makes it difficult for the model to obtain global feature information. What’s more, ViT calculates self-attention for all patches in the entire feature layer, so it is difficult to collect global feature information for images with high-resolution pixels. To solve the above problems, Liu Z et al. proposed the Swin Transformer model [38], as shown in Fig 1. The Swin Transformer starts with the smallest image patch, increases the down-sampling multiples layer by layer, and merges adjacent feature blocks in deeper feature layers to build a multi-level feature map. Meanwhile, the Swin Transformer confines the computational self-attention to a fixed window, and transfers and interacts with features in different windows through a shifted window operation to obtain global feature information.

To sum up, Swin Transformer is based on ViT by introducing hierarchical design and moving sliding window operation, so that Swin Transformer can be used as a general backbone network in computer vision tasks such as object detection and strength segmentation.

**Fig 2:**
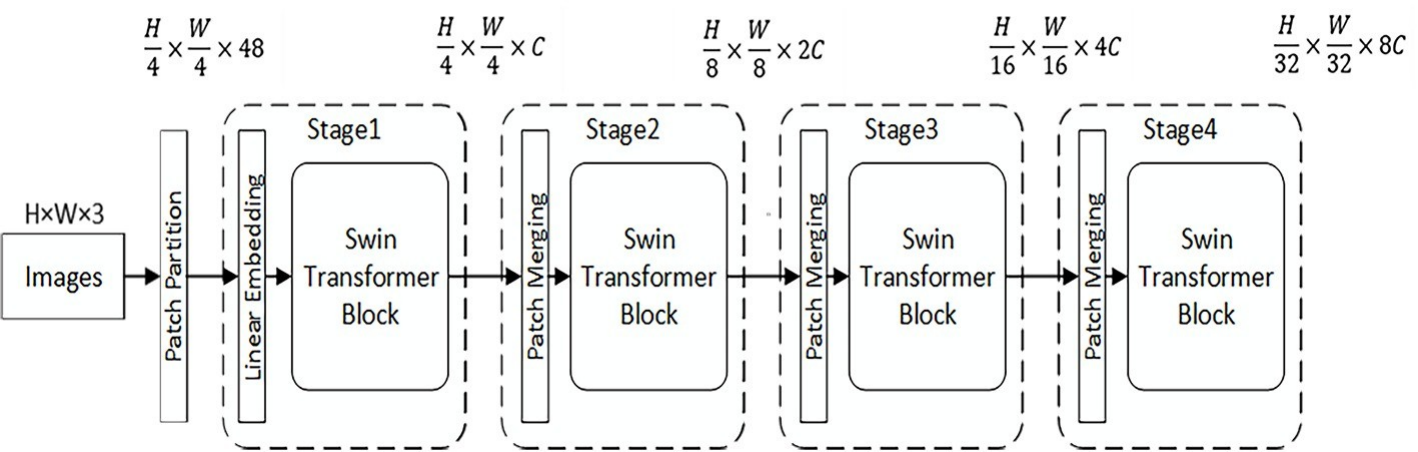
Swin Transformer structure.

The overall architecture of the Swin Transformer is shown in Fig 2. The specific process is: 1) First, the RGB image with the input size of H × W × 3 is split into equal size nonoverlapping image blocks (patches) through the patch partition module (Patch Partition) module, and the size of each image block is 4 × 4, and the number of channels C = 3, its feature dimension is 4 × 4 × 3 = 48, and the input image is divided into H/4×W/4 image blocks. 2) Second, in stage1, the Linear Embedding layer projects the feature dimension of each image block to any dimension C, and then sends it to the Swin Transformer Block to perform self-attention calculation on the input features. 3) To generate a hierarchical feature layer, two adjacent 2 × 2 image blocks are spliced through the image block merging layer (Patch Merging), so that the number of image blocks becomes H/8 × W/8, and at the same time, the dimension is expanded by a factor of 4, which is 4 C. Then, a convolutional network is used to reduce the dimensionality of the spliced 4 C-dimensional features. Therefore, the entire stage 2 reduces the number of image blocks by a factor of 4, and the feature dimension becomes 2 C. 4) The operation of stage 3 and stage 4 is the same as that of stage 2, which reduces the number of input features by 4 times and expands the feature dimension by 2 times, so that the entire feature extraction module generates a hierarchical feature layer.

Compared with the Vision Transformer, the Swin Transformer replaces the standard multi-head self-attention module (MSA) with a window-based multi-head self-attention module (W-MSA) and a shifted windows multi-head self-attention (SW-MSA), realizing the transfer and interaction of features in different windows.

**Fig 3:**
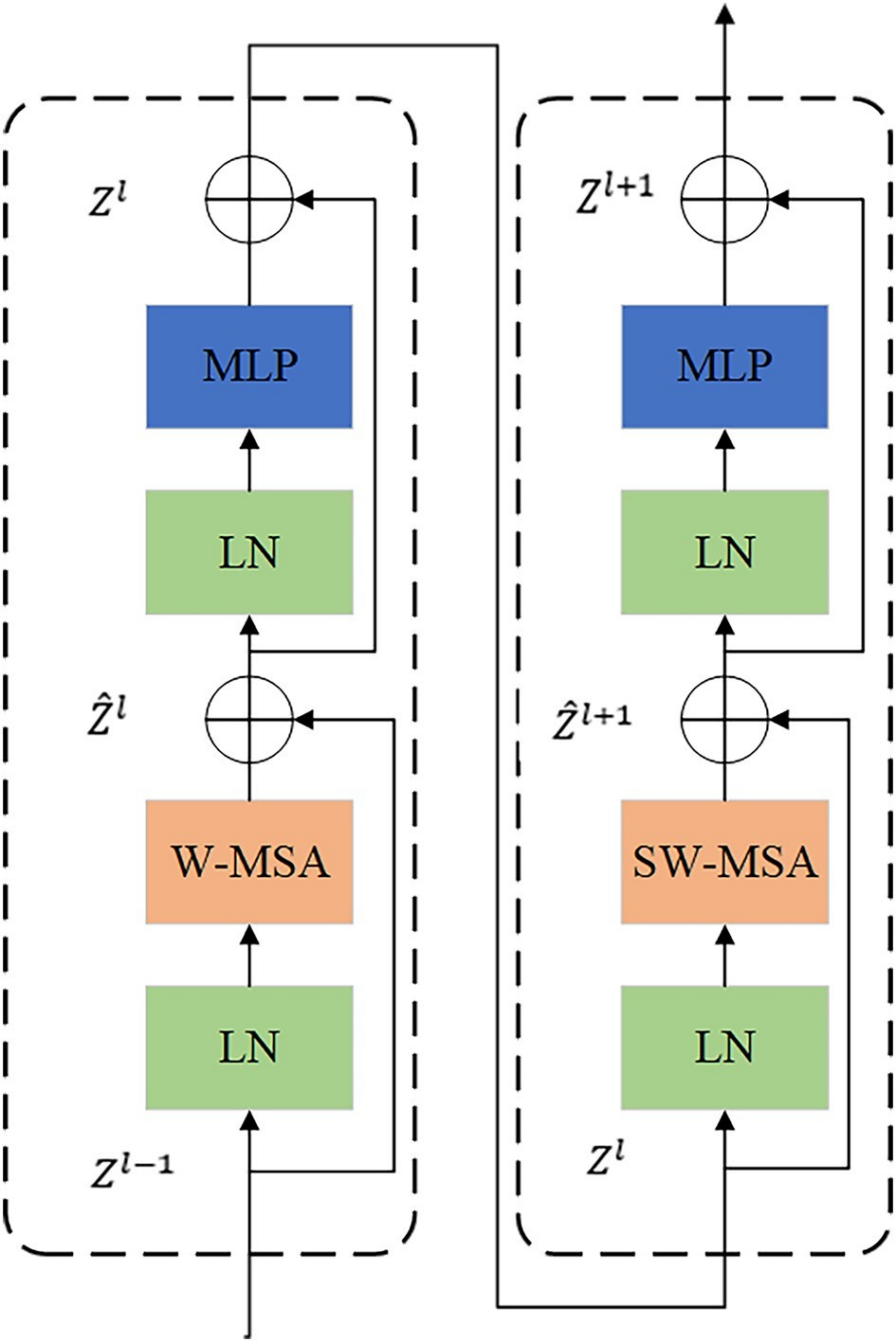
Two consecutive swin transformer blocks.

Fig 3 shows two consecutive Swin Transformer Blocks, including a window-based multi-head self-attention module (W-MSA) and a moving-window-based multi-head self-attention module (shifted windows multi-head self -attention, SW-MSA), so as to realize the transfer and interaction of image block features in different windows. Where ${\hat{Z}}^{l}$ land *Z*^*l*^ represent the output features of the (S)W-MSA module and the multilayer perceptron (MLP) module output features of the lth block, respectively. Meanwhile, a normalization layer (Layer-Norm LN) is used before each MSA module and MLP modules for normalization, and a residual connection is used after each MSA and MLP. Therefore, the calculation process of two consecutive Swin Transformer blocks can be expressed as:

$${\hat{Z}}^{l}=W-MSA\left(LN\left(Z^{l-1}\right)\right)+Z^{l-1}$$ (1)

$$Z^{l}=MLP\left(LN\left({\hat{Z}}^{l}\right)\right)+{\hat{Z}}^{l}$$ (2)

$${\hat{Z}}^{l+1}=SW-MSA\left(LN\left(Z^{l}\right)\right)+Z^{l}$$ (3)

$$Z^{l+1}=MLP\left(LN\left({\hat{Z}}^{l+1}\right)\right)+{\hat{Z}}^{l+1}$$ (4)

### Self-attention based on moving windows

The Vision Transformer model uses global self-attention when performing image classification tasks, that is, calculating the relationship between each token and all other tokens (Attention Map). For computer vision tasks such as object detection and power segmentation that need to process high-pixel resolution images, performing global self-attention will undoubtedly bring a huge amount of computation. In response to this problem, the Swin Transformer proposes to establish a nonoverlapping local window, calculate self-attention in this window, replace global attention, and then use the movement of the window in SW-MSA to realize the connection and interaction of image block features in different windows.

**Fig 4:**
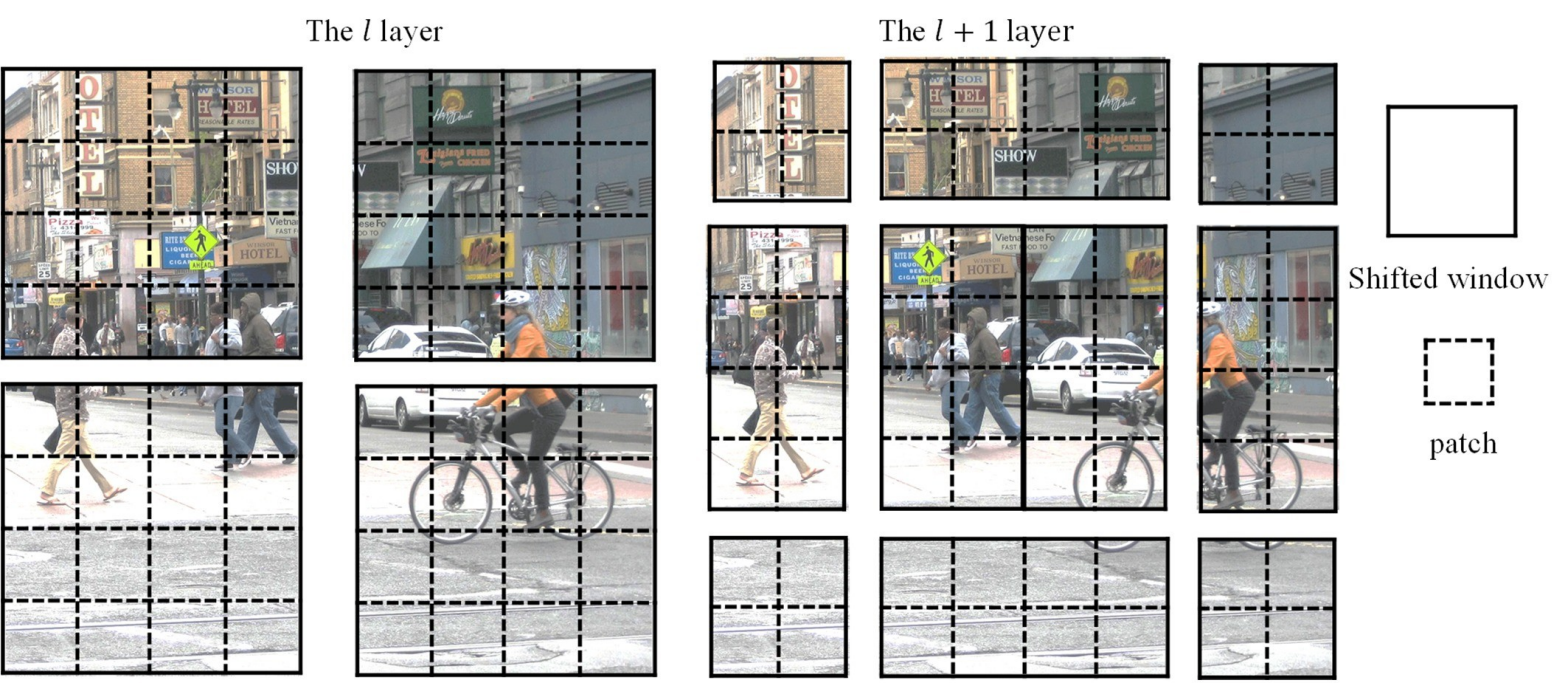
Self-attention computed based on shifted windows.

As shown in Fig 4, in the l layer, the Swin Transformer first utilizes W-MSA, that is, it establishes a regular local window and partitions the image, and then computes self-attention in each window. Assuming that the picture has a total of h × w patches, and each window contains N×N patches, the computational complexity of W-MSA can be expressed as follows:

$$\Omega (W-MSA)=4hwC^{2}+2N^{2}hwC$$ (5)

Since the number of patches in the window is much smaller than the number of patches in the picture, when the window size is fixed, the computational complexity of W-MSA is linearly related to the picture size.

In the l+1 layer, using SW-MSA, the window is moved down to right, a new window is formed, and the self-attention calculation is performed in the new window. The new window contains unrelated patches in the l^th^ layer, in order to realize the communication and transfer of image block features in different windows.

**Fig 5:**
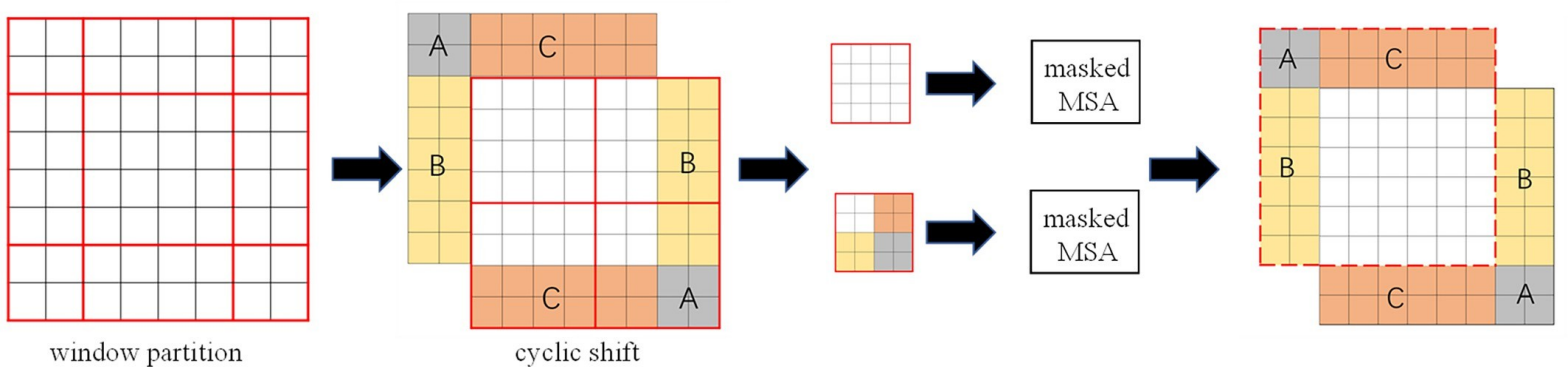
Calculation of self-attention of moving windows based on cyclic shifting.

However, after the window is moved, the number of windows will increase. In order to solve the problem of increasing the number of windows, Swin Transformer proposes a more efficient self-attention calculation method, namely the cyclic shift to the upper left. As shown in Fig 5, after the shifted window moves to the lower left corner, there are three incomplete windows. By shifting the three sub-windows A, B, and C in the upper left, three windows that can perform self-attention calculation are formed. However, these three windows are composed of several non-adjacent sub-windows, so a masked mechanism is required to limit the self-attention to the sub-windows for calculation, preventing non-adjacent image blocks from performing self-attention calculation. After the self-attention calculation in the window is completed, the cyclically displaced sub-window is restored. After these operations, the self-attention calculation results are obtained under the condition of maintaining the original number of windows, and the transfer and interaction of adjacent image block features are realized.

### Swin-YOLOv4 algorithm design

**Fig 6:**
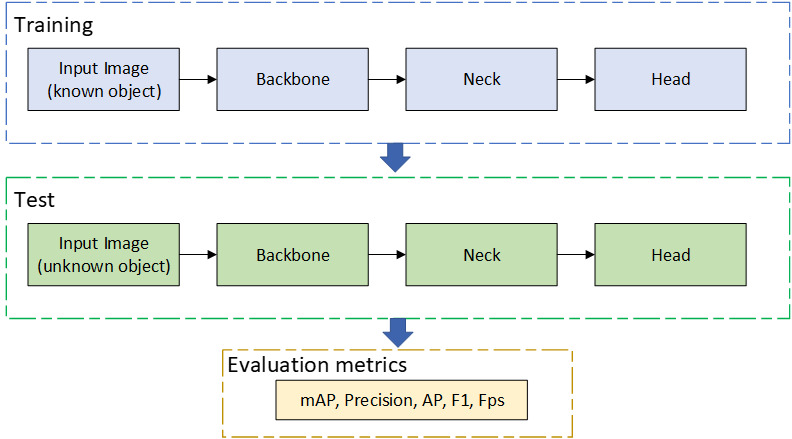
Abstract view of object detection systems.

The working principle of SwinT-YOLOv4 is shown in Fig 6. In the training process, known pictures are first input, and the training is completed by backbone, neck, and head modules. In the test process, unknown pictures are input, and the test is also completed by backbone, neck, and head modules. Finally, a series of evaluation metrics is output.

**Fig 7:**
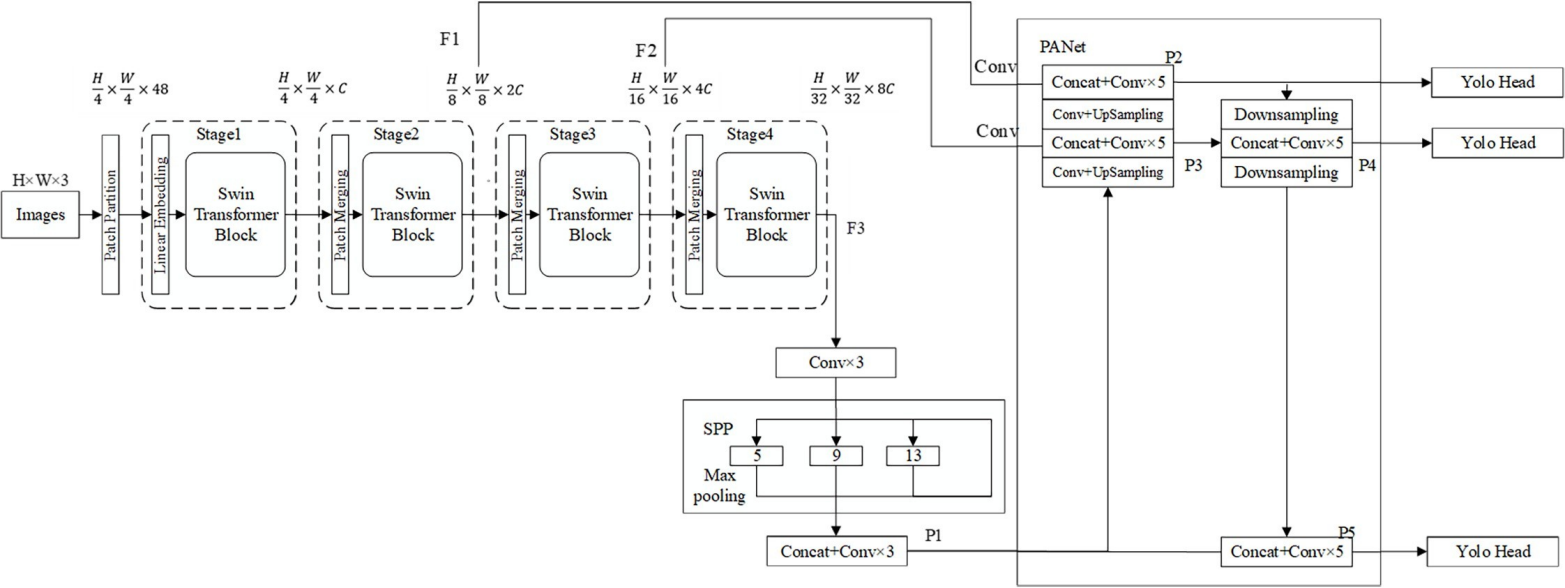
SwinT-YOLOv4 structure.

The backbone in YOLOv4 based on CNN is replaced by the Swin Transformer in the proposed algorithm. The feature-fusing neck and predicting head of YOLOv4 is remained. The overall architecture of SwinT-YOLOv4 is shown in Fig 7. The architecture is mainly divided into Swin Transformer backbone network, enhanced feature extraction network combined with SPP and PANet, and Yolo Head feature prediction operation. The specific process is: 1) Extract the feature layers output by stage2, stage3, and stage4 in the Swin Transformer model, and set them as F1, F2, and F3 respectively. The size and dimension of the feature maps of the three feature layers are 28 × 28 × 192, 14 × 14 × 384 and 7 × 7 × 768. 2) Perform a convolution operation on the feature layers F1 and F2 and input them into the PANet network. The F3 feature layer first performs three convolution operations and then inputs the SPP network to perform the pooling operation. The output results of the SPP network are stacked and convolved three times to form the feature layer P1, and finally, the feature layer P1 is input to the PANet network. 3) The PANet network enhances the extraction of the feature layer features through feature stacking, up-sampling and down-sampling, and convolution operations, and outputs three higher-quality effective feature layers. 4) Perform the Yolo Head operation on the three effective feature layers output by the PANet network, that is, perform a 3×3 convolution on the effective feature layer to complete the integration of the features, and then perform a 1×1 convolution to use the acquired features to obtain the prediction result.

**Fig 8:**
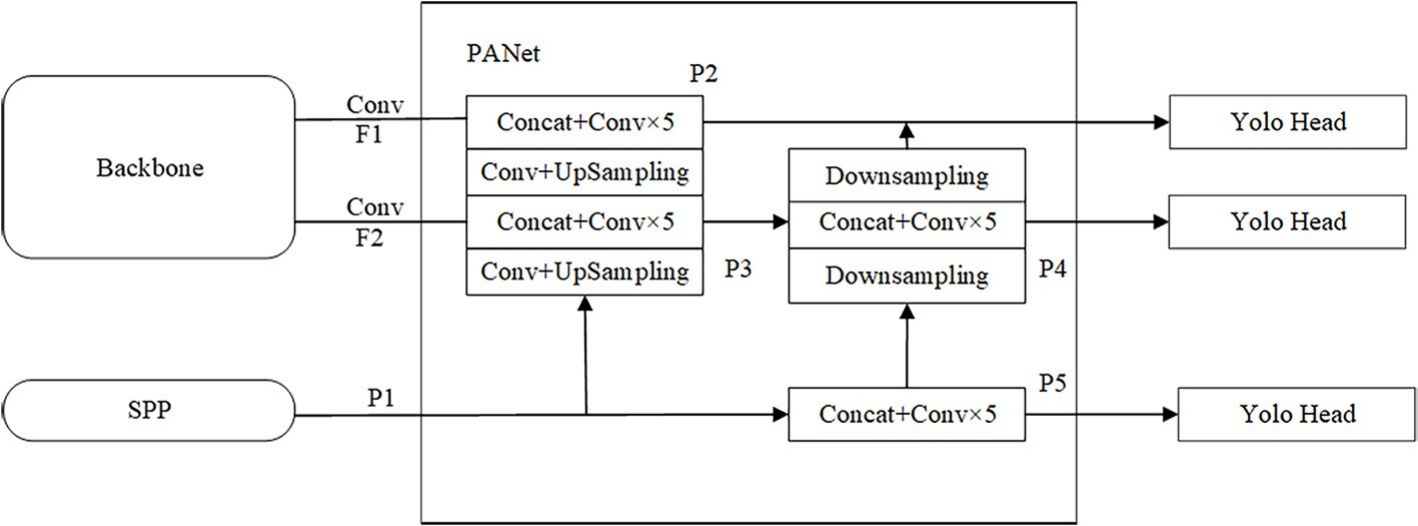
SPP network structure.

Fig 8 shows the structure of the SPP network. The specific process of the network is as follows: After the output of the feature layer stage4 in the Swin Transformer model completes the three convolution operations, it is inputted to the SPP network. After entering the SPP network, four different scales of maximum pooling are used for processing. The total pool kernel sizes for the maximum pooling are 13 × 13, 9 × 9, 5 × 5, and 1 × 1 (1 × 1 means no processing). Finally, the feature stacking and three convolution operations are performed on the output feature layer to obtain the P1 feature layer for further feature extraction.

**Fig 9:**
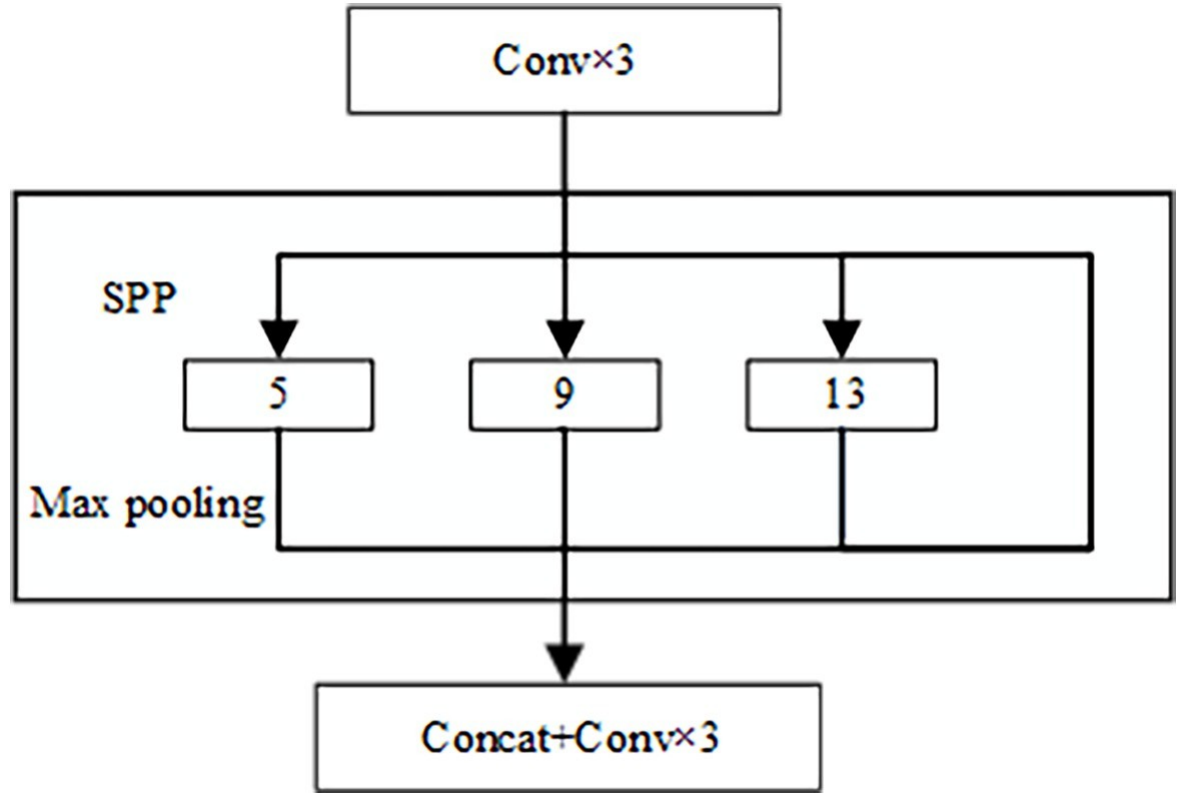
PANet network structure.

The network structure of PANet is shown in Fig 9. PANet is an instance segmentation algorithm. In the traditional feature pyramid structure, the network first completes the up-sampling feature extraction from the bottom up, and then completes the down-sampling feature extraction from the top down, so as to realize the repeated feature extraction. Get higher-quality feature layers. In the SwinT-YOLOv4 model, the feature layer F1 and F2 output by the Swin Transformer backbone network are mainly used on the feature layer F3 output by the SPP network to use the PANet network to enhance the feature extraction. The specific process is: 1) First, the feature layer P1 performs one convolution and up-sampling operation and performs feature stacking and five convolution operations with the feature layer F2 that has completed one convolution operation to obtain the feature layer P3. Then perform a convolution and up-sampling operation on the feature layer P3, and perform feature stacking and five convolution operations with the feature layer F1 that has completed a convolution operation to obtain a feature layer P2. 2) Perform a down-sampling operation on the feature layer P2, and then perform feature stacking and five convolution operations with the feature layer P3 to obtain the feature layer P4. 3) Perform the down-sampling operation on the feature layer P4, and then perform the feature stacking and five convolution operations with the feature layer P1 to obtain the feature layer P5. After these operations, enhanced feature extraction is achieved through the PANet network and three more efficient effective layers of features P2, P4 and P5 are obtained. Finally, the Yolo Head operation is performed on these three effective feature layers to obtain the prediction result.

## Results

### Experimental environment

The deep learning framework used in this paper is Pytorch1.7.0, and the experimental environment is Python3.8 and CUDA11.0. All model training and testing are performed on the CPU of AMD EPYC 7543 32-CORE Processor 15-core memory 80GB, and the GPU of RTX3090 with 24GB of video memory. It is carried out on the server. The pictures detected in the experiment come from the test set in the dataset.

The dataset used in this experiment is from the COCO2017 dataset. A total of 12000 pictures of car and person in the COCO2017 dataset are extracted and converted into the VOC dataset format through Python 3.8. The training set, the validation set, and the test set are divided according to the 8:1:1 ratio. The researchers of Swin Transformer completed the model training based on ImageNet and obtained the network pre-training weight file. In order to improve the training efficiency, this experiment chose to conduct model training on the basis of the above-mentioned pre-training weight file. The optimizer adopts SDG, the learning rate descending method adopts cos, and other training parameters are shown in Table 1.

**Table 1:** Setting of training parameters.

Input image	Initial learning rate	Weight decay	Batch size	Epochs
416×416	1e-2	5e-4	16	200

### Loss function curve

**Fig 10:**
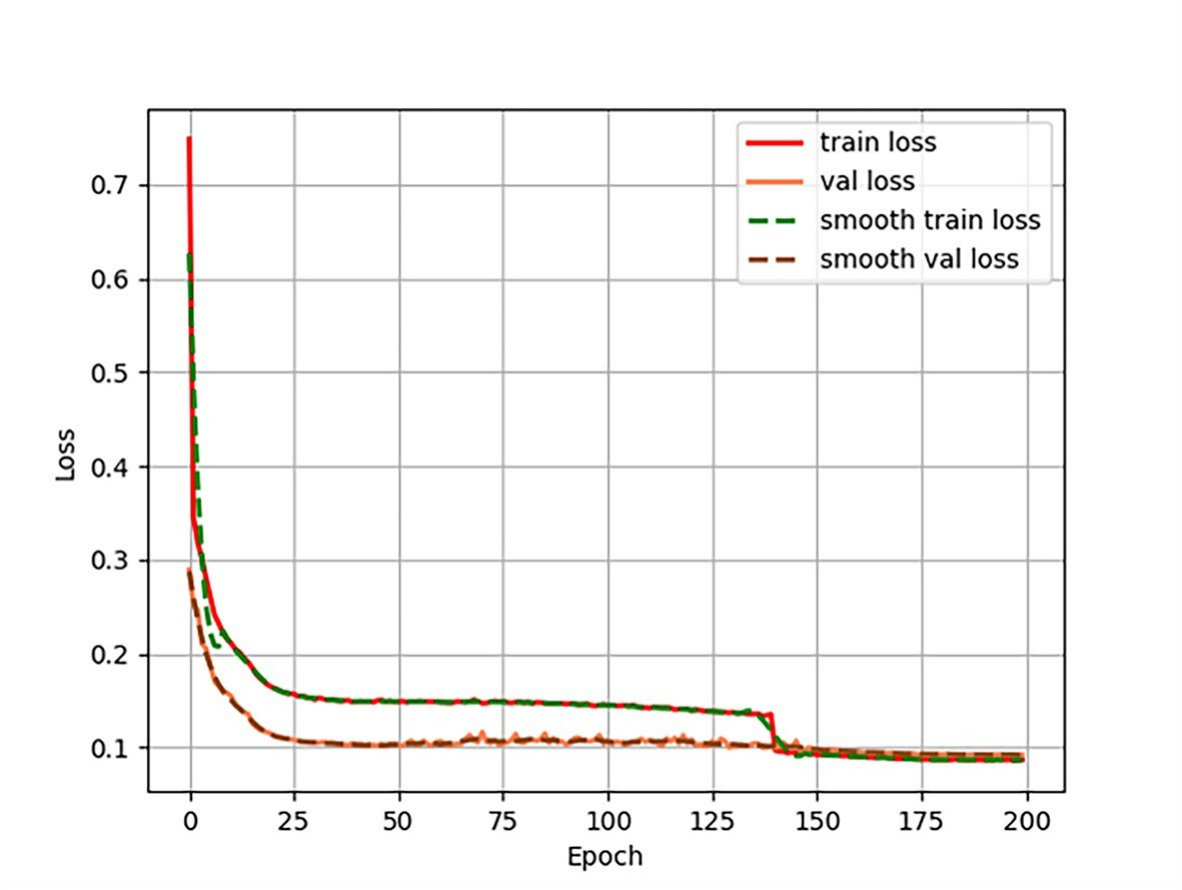
SwinT-YOLOv4 loss function curve.

The loss function used in this algorithm is CioU Loss. CioU_Loss takes into account overlap area, aspect ratio, and center point distance. The loss function image of the SwinT-YOLOv4 model training is shown in Fig 10. It can be seen from the figure that the feature extraction training is performed on the basis of the pre-training weights, and the loss value converges quickly. After about 30 iterations, the loss value of the training set (train loss) and the loss value of the validation set (val loss) tend to be stable. After 100 iterations, the train loss is stable at around 0.146, the value loss is stable at around 0.104, and the parameter convergence is good. Since the improved SwinT-YOLOv4 algorithm performs multi-scale feature fusion and obtains a higher-quality feature layer by strengthening the feature extraction network, in order to avoid overfitting due to too many iterations, the training is stopped after 200 rounds of training, and the final result is obtained, train loss is stable at around 0.087, val loss is stable at around 0.092.

### Performance analysis

Before performing model evaluation and comparison, appropriate evaluation indicators need to be determined. In the article, the experimental results are comprehensively measured from the four aspects of detection precision (Precision), average precision (Average Precision), F_1_ (harmonic mean of precision and recall), and mAP (average mean precision).

The formula for calculating precision is the following:

$$\;precision\;=\frac{TP}{TP+FP}\times 100\text{\%}$$ (6)

The recall rate refers to the calculation formula as follows:

$$\;recall\;=\frac{TP}{TP+FN}\times 100\text{\%}$$ (7)

The formula for calculating F_1_ is the following:

$$F_{1}=\frac{2\times \;precision\;\times \;recall\;}{\;precision\;+\;recall\;}$$ (8)

The average precision rate refers to the area of the curve enclosed by the precision rate and the recall rate, so the calculation formula of AP is the following:

$$AP=\int_{0}^{1}precision(recall\;)d(precision\;)$$ (9)

The formula for calculating the mean average accuracy rate (mAP) is the following:

$$mAP=\sum_{i=1}^{N}\frac{AP_{I}}{N}(N=2)$$ (10)

In the above formula, TP refers to the number of correct detections in the detection object, FP refers to the number of false detections in the detection object, and FN refers to the number of missed detection objects in the detection object. When the iou(intersection over union) value of the predicted sample is greater than 0.5, it is judged as correct prediction. N refers to the number of categories for object detection. High precision indicates that the model has high accuracy in judging the object category when performing the object detection task, and a high recall rate indicates that the model misses a small number of objects when performing the object detection task. F_1_ is a measure of combined precision and recall. The mean mAP average precision rate is usually used to evaluate the accuracy of an algorithm’s object detection. The higher the mAP value, the better the object detection effect of the algorithm.

The precision of the YOLOv4 and the SwinT-YOLOv4 are shown in Table 2. The object detection precision of SwinT-YOLOv4 for cars is 89.04%. The object detection precision of person is improved by 4.41% compared with YOLOv4, reaching 94.16%. The AP of SwinT-YOLOv4 for cars and person increases by 5.63% and 4.62%, respectively. Meanwhile, the value of F_1_ has decreased slightly. It can be concluded that the improved SwinT-YOLOv4 has a significant improvement in object detection accuracy and improves the problem of false detection, but compared with the YOLOv4, the proportion of missed reports of the model have a certain increase.

**Table 2:** Comparison of YOLOv4 and SwinT-YOLOv4 results.

Model	Objects	Precision	AP	F_1_
**YOLOv4**	**Cars**	89.95%	64.73%	0.61
	**Person**	89.75%	67.37%	0.65
**SwinT-YOLOv4**	**Cars**	89.04%	70.36%	0.56
	**Person**	94.16%	71.99%	0.56

**Fig 11:**
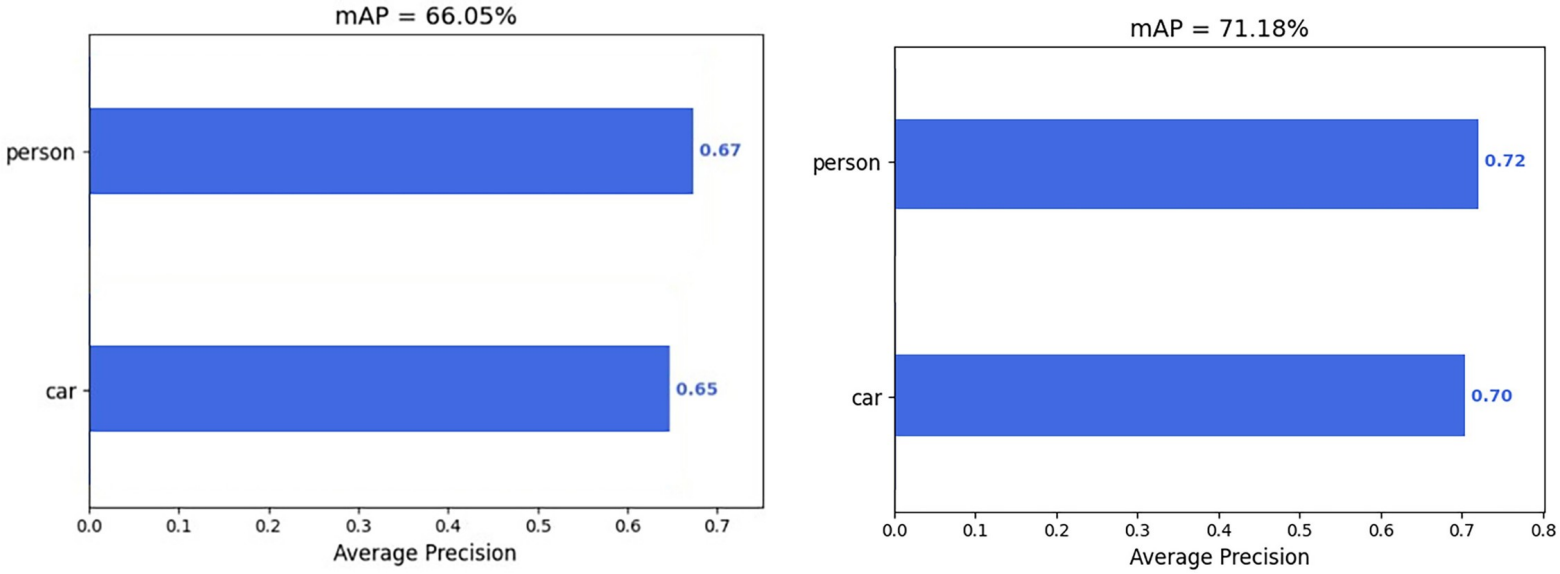
mAP of SwinT-YOLOv4 and YOLOv4. (A) mAP of YOLOv4; (B) mAP of SwinT-YOLOv4.

The mAP values of the SwinT-YOLOv4 and the YOLOv4 are shown in Fig 11. The mAP of the improved SwinT-YOLOv4 is increased by 5.13% to 71.18%. This experiment shows that the SwinT-YOLOv4 model with Swin Transformer as the backbone network can greatly improve the object detection effect of the model in traffic scenes by strengthening feature extraction and obtaining higher-quality feature layers, and can accurately detect the vast majority of objects in the picture.

Floating point of operations (FLOPs) refers to the number of floating-point operations, which is used to measure the complexity of the model. The larger the value is, the higher the complexity of the model is. The Table 3 shows that the SwinT-YOLOv4 has larger FLOPs, so the complexity of the model is higher. Frames per second (Fps) refers to the number of frames per second that the model transmits when processing video files. Therefore, the Fps value is used as the evaluation index to evaluate the detection speed of the model when it performs object detection tasks. The video detection of the YOLOv4 and the SwinT-YOLOv4 models is performed on a server with CPU i5-7300HQ and graphics card GTX1050, respectively, and the detection speed of YOLOv4 is about 6.31 Fps, and the detection speed of SwinT-YOLOv4 is about 5.21 Fps. It can be concluded that compared with the YOLOv4 algorithm, the improved SwinT-YOLOv4 has roughly the same processing speed but slightly lower, so there is still some room for improvement in the detection speed index of SwinT-YOLOv4.

**Table 3:** Comparison of Fps and FLOPs between YOLOv4 and SwinT-YOLOv4.

Model	Fps	FLOPs
YOLOv4	6.31	3.01×10^10^
SwinT-YOLOv4	5.21	4.33×10^10^

**Fig 12:**
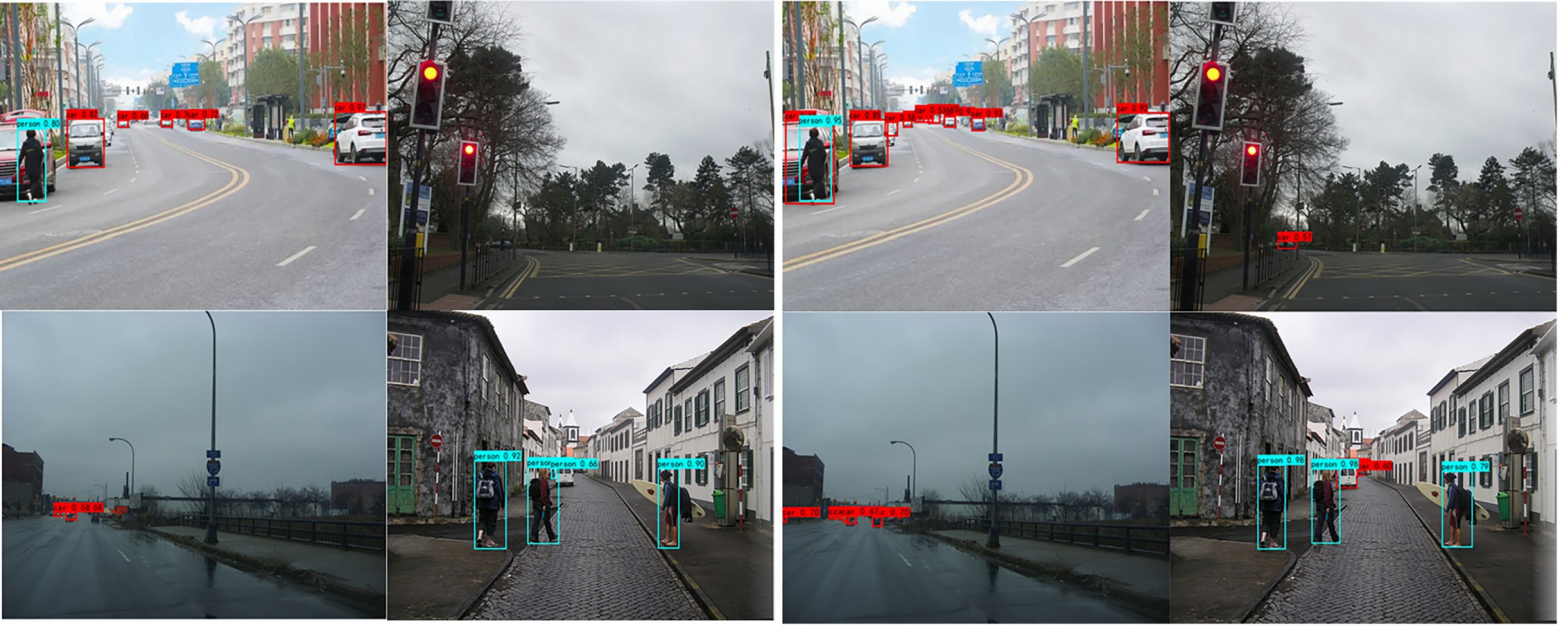
Comparison of the detection effect of SwinT-YOLOv4 and YOLOv4. (A) Detection effect of YOLOv4; (B) Detection effect of SwinT-YOLOv4.

Fig 12 shows the detection effect of the pre-improved YOLOv4 model and the improved SwinT-YOLOv4 model on some samples in the test set, respectively. Compared with the original model, the backbone network Swin Transformer of the improved SwinT-YOLOv4 model improves the quality of feature extraction, and uses a multi-scale feature fusion mechanism to obtain higher quality feature layers. Therefore, it can be seen from Fig 12 that in the case where SwinT-YOLOv4 is used, the confidence score for object detection is significantly improved, the positioning effect is more accurate, and the detection accuracy is significantly improved. The accuracy and detection rate are significantly improved when the object is blurred, the small object, the weather is dark, and the detection objects are occluded by each other. Therefore, the improved Swin Transformer model performs better in object detection tasks.

## Discussion

The precision of SwinT-YOLOV4 object detection algorithm for cars and person has reached 89.04% and 94.16% after 200 epochs of training on 12000 images. The mAP value reached 71.18%, which was 5.13% higher than before improvement. Meanwhile, the average precision of cars and person is increased by 5.43% and 4.62%, respectively. Through the test and comparison of the images, it can be clearly concluded that the SwinT-YOLOv4 has significantly improved the detection ability and detection results for object occlusion, small objects and bad weather. However, the value of SwinT-YOLOv4’s FLOPs is slightly increased, indicating that the improved model is slightly more complex than YOLOv4. Meanwhile, F_1_ value and Fps value are slightly lower than those of the algorithm before improvement, indicating that although the SwinT-YOLOv4 improves the detection accuracy, the model has the problem of missing detection and detection speed still need to be further improved.

The performance of the object detection model SwinT-YOLOv4 is compared with other advanced models, and the results are shown in Table 4. It is not difficult to find that our model has obvious advantages in object detection performance in traffic scenes compared with other algorithms.

**Table 4:** The performance comparison of existing model.

Model	Object	Average Precision
SwinT-YOLOv4	Cars, Person	91.6%
[8]	Pascal VOC	63.4%
[34]	Person, Bus, Cars, Rider, Truck, Bike, Motor	40.6%
[39]	Person, Bike, Cars	89.6%
[40]	Pedestrian	90%

Although the SwinT-YOLOv4 model has high accuracy and reliability of target detection in traffic scenarios, there are still some defects. First of all, the experimental results show that the value of F_1_ decreases slightly, so the proportion of missed reports of our model have a certain increase. At the same time, in the traffic scene with complex road conditions, the real-time and detection speed of target detection have high requirements, but the detection speed of our model is slightly lower. Therefore, overcoming the shortcomings mentioned above is an important direction of future research.

## Conclusion

This paper focuses on the object detection of vehicles and pedestrians in traffic scenes and focuses on the improved SwinT-YOLOv4 object detection algorithm based on YOLOv4. The main contents and the contribution of the paper are as follows:

1. The overall architecture of the SwinT-YOLOv4 model is studied and analyzed, the feature extraction process of the model is described in detail, and the principle of the enhanced feature extraction network SPP and PANet network of the model is introduced in detail.
2. The configuration of the experimental environment, the setting of training parameters, and the preparation of the dataset are introduced, and the improved SwinT-YOLOv4 is used to train the dataset. Then, the performance of SwinT-YOLOv4 and YOLOv4 was tested in four aspects: Precision, average precision, F_1_ and mAP. The experimental results show that the detection accuracy of the improved SwinT-YOLOv4 for vehicles and pedestrians reaches 89.04% and 94.16%, respectively, and the detection performance is greatly improved, which can better complete the object detection task of traffic scenes.

In the future, we will focus on improving the detection speed of the algorithm and reducing the proportion of missed reports of the algorithm for further research. At the same time, we will expand our dataset of experiments, adding traffic lights and more types of vehicles such as trucks, motorcycles and bicycles to make our research more comprehensive.

## Supporting information

S1 File Dataset. (DOCX)
