# Supplementary material for: An object detection algorithm combining self-attention and YOLOv4 in traffic scene
Source: PLoS One. 2023 May 18;18(5):e0285654. doi: 10.1371/journal.pone.0285654 (PMC10194927; doi:10.1371/journal.pone.0285654)
Supplement: S1 File — (DOCX) [file pone.0285654.s001.docx]

In our experiment, we extracted pictures about traffic scene targets from COCO data set to carry out the experiment.

The datasets can be downloaded from the below links: COCO: Datasets https://cocodataset.org/#home.
